# xCT (SLC7A11) expression confers intrinsic resistance to physical plasma treatment in tumor cells

**DOI:** 10.1016/j.redox.2019.101423

**Published:** 2020-01-03

**Authors:** Sander Bekeschus, Sebastian Eisenmann, Sanjeev Kumar Sagwal, Yana Bodnar, Juliane Moritz, Broder Poschkamp, Ingo Stoffels, Steffen Emmert, Muniswamy Madesh, Klaus-Dieter Weltmann, Thomas von Woedtke, Rajesh Kumar Gandhirajan

**Affiliations:** a Leibniz Institute for Plasma Science and Technology (INP Greifswald), ZIK Plasmatis, Felix-Hausdorff-Str. 2, 17489, Greifswald, Germany; b Greifswald University Medical Center, Department of General, Visceral, Thoracic and Vascular Surgery, 17475, Greifswald, Germany; c University Hospital Essen, Department of Dermatology, Venereology, and Allergology, University of Duisburg-Essen, 45122, Essen, Germany; d Rostock University Medical Center, Clinic for Dermatology and Venereology, Strempelstr. 13, 18057, Rostock, Germany; e Institute for Hygiene and Environmental Medicine, Walther-Rathenau-Str. 48, 17489, Greifswald, Germany; f Center for Precision Medicine, Department of Medicine, University of Texas Health San Antonio, San Antonio, TX, USA

**Keywords:** Cancer, Glutathione, kINPen, Melanoma, Plasma medicine

## Abstract

Cold physical plasma is a partially ionized gas investigated as a new anticancer tool in selectively targeting cancer cells in monotherapy or in combination with therapeutic agents. Here, we investigated the intrinsic resistance mechanisms of tumor cells towards physical plasma treatment. When analyzing the dose-response relationship to cold plasma-derived oxidants in 11 human cancer cell lines, we identified four ‘resistant’ and seven ‘sensitive’ cell lines. We observed stable intracellular glutathione levels following plasma treatment only in the ‘resistant’ cell lines indicative of altered antioxidant mechanisms. Assessment of proteins involved in GSH metabolism revealed cystine-glutamate antiporter xCT (SLC7A11) to be significantly more abundant in the ‘resistant’ cell lines as compared to ‘sensitive’ cell lines. This decisive role of xCT was confirmed by pharmacological and genetic inhibition, followed by cold physical plasma treatment. Finally, microscopy analysis of *ex vivo* plasma-treated human melanoma punch biopsies suggested a correlation between apoptosis and basal xCT protein abundance. Taken together, our results demonstrate that xCT holds the potential as a biomarker predicting the sensitivity of tumor cells towards plasma treatment.

## 1 Introduction

Among many types of solid tumors, breast, colorectal, lung, and prostate account for about 60% of all cancer cases in the western world. The extent of cancerous disease is set to increase by up to 18.2 million in 2020. With sophisticated treatment options emerging at ever-higher costs, policymakers face difficult decisions about therapy coverage and access to treatment [1]. Treatment of solid tumors mostly relies on traditional options such as surgery, radiation, and chemotherapy. Recent advances in targeted therapy led to the development of novel therapeutics that target specific dysregulated pathways in tumors, hence offering alternative treatment options.

A majority of existing strategies (e.g., radiotherapy, chemotherapy, and small molecule therapy) targeting tumor cells eventually induce oxidative stress and cell death. Cold physical plasma is an emerging treatment strategy, which employs unique RONS (reactive oxygen and nitrogen species) to selectively target tumor cells by perturbing cellular redox balance leading to oxidative stress, mitochondrial dysfunction, and cell death. The advantage of this strategy is that it can directly transfer RONS to the tumor tissue without the need for any systemic agents. Physical plasma consists of multiple components such as electrical fields, ions and electrons, thermal and UV radiation, RONS, and visible light [2], leading to endoplasmic reticulum (ER) and mitochondrial dysfunction, and subsequently to apoptosis [[3], [4], [5]]. RONS identified to be critical for plasma-mediated effects are atomic and singlet delta oxygen [[6], [7], [8]], hydrogen peroxide [[9], [10], [11]], nitrite [[12], [13], [14]], nitric oxide [[15], [16], [17]], and hydroxyl radicals [[18], [19], [20]]. Recently, we demonstrated that plasma treatment increased the expression of SLC22A16 in tumor cells, which led to an intracellular accumulation of anthracyclines resulting in synergistic tumor toxicity [21]. However, tumors are extraordinarily heterogeneous, and treatment responses are determined frequently by the cellular origin and mutational load.

Most of the current resistance mechanisms are identified from traditional chemotherapies and targeted small molecules therapies, and this knowledge can help to anticipate the resistance mechanisms of emerging cold plasma-based therapies. Intrinsic resistance is defined as innate resistance that exists before the patient is administered with drugs due to pre-existing somatic mutations [22], heterogenic tumor stem cell populations [23], and activation of intrinsic efflux pathways [24] used as a defense against anti-cancer drugs. However, there are no studies available on the intrinsic resistance mechanisms towards cold physical plasma treatment in tumor cells. In the current investigation, we address this question in multiple tumor cell lines and identified xCT (SLC7A11) as a putative predictor for resistance against cold plasma treatment in tumor cells.

## 2 Materials and methods

### 2.1 Cell culture

The human tumor cell lines SK-MEL-28, MeWo, MaMel86a, Panc-1, Miapaca2GR, HeLa, MDA-MB-231, PC-3, 501-Mel, OVCAR3, and A375, as well as non-malignant human HaCaT keratinocytes, were cultured in high glucose Dulbecco Minimum Essential Media or Roswell Park Memorial Institute media (DMEM, RPMI; Invitrogen) supplemented with 10% fetal calf serum (FCS), 1% penicillin/streptomycin, and 1% glutamine. For 2D culture assays, 1 × 10^4^ cells/well were incubated in 96-well culture plates (Nunc) in complete cell culture medium 16 h before their experimental use. In some experiments, cells were pretreated with azacytidine (AZA), vorinostat (VOR), sulfasalazine (SFL), ezatiostat (EZA), and butathione sulfoximine (BSO) (all Sigma Aldrich) for 16 or 24 h as indicated. The atmospheric pressure argon plasma jet kINPen (neoplas tools) served as a RONS-generating source and was operated at a frequency of 1 MHz with a feed gas flux of 2 L per minute of argon gas (99.9999% purity; Air Liquide). Argon gas (plasma off) treated medium served as control throughout all experiments.

### 2.2 Metabolic activity and cell viability

To assess metabolic activity, 1 × 10^4^ cells were plated in 96-well culture plates (Nunc) in complete DMEM. Sixteen hours later, cells were challenged with 30, 60, and 120 s of plasma treatment before further incubation for 6 h or 24 h. Subsequently, wells were loaded with 100 μM of resazurin (Alfa Aesar) that is transformed to fluorescent resorufin by metabolically active cells. The plate was incubated for 2 h at 37 °C, and fluorescence was measured using a multimode plate reader (Tecan) at *λ*_ex_ 535 nm and *λ*_em_ 590 nm. Metabolic activity was shown as percent of the untreated control. To determine toxicity, cells were loaded with sytox orange (1 μM; Thermo Fisher) for 30 min at 37 °C. Cells were imaged with a 20x objective using a live cell high throughput imaging system (Operetta CLS; PerkinElmer). Algorithm-based quantitative image analysis was performed using dedicated software (Harmony 4.8; PerkinElmer).

### 2.3 Western blotting

Cells were harvested in ice-cold PBS and lysed in RIPA buffer (Cell signaling) supplemented with complete protease and phosphatase inhibitors (PIM complete; Roche) for 20 min on ice. Fifty millimolar of N-Ethylmaleimide (NEM; Sigma) was supplemented for s-glutathionylation preparations. After centrifugation at 15,000×*g* for 15 min at 4 °C, total protein in whole-cell extracts was quantified using Rotiquant (Carl Roth). Forty micrograms of protein were resolved by SDS-PAGE (Invitrogen) and blotted on PVDF membranes (Invitrogen). The membranes were probed with anti-GSTP1, anti-xCT, anti-catalase, anti-SOD1, anti-GPX1, anti-γGCS, or anti-β actin (Santa Cruz) primary antibodies followed by secondary horse-radish peroxidase (HRP) coupled antibodies (Santa Cruz). Signals were acquired in a chemiluminescence detection system (Applied Biosystems) in a linear dynamic range.

### 2.4 Quantitative real-time PCR

Total mRNA was isolated using a RNA isolation kit (BioSell GmbH). One microgram of mRNA was converted to cDNA using the PrimeScript cDNA synthesis kit (Takara Bio). Predesigned *KiCqStart SYBR Green* primers for human β-actin (Fwd: GATGGGCGGCGGAAAATAG Rev: GCGTGGATTCTGCATAATGGT) and SLC7A11 (Fwd: CCTCTATTCGGACCCATTTAGT Rev: CTGGGTTTCTTGTCCCATATAA) were obtained from Sigma-Aldrich. qPCR assays were carried out using *Power SYBR Green* PCR Master Mix in a Quantstudio 1 device (ThermoFisher) with 40 cycles of PCR amplification using 95 °C for 30s, 95 °C for 5s, and 60 °C for 30s for each cycle. The ΔΔCt method was employed to calculate fold changes in gene expression using the Quantstudio design and analysis software.

### 2.5 Determination of cellular glutathione

Total and oxidized glutathione in tumor cells was determined from 1 × 10^4^ cells at 6 h following plasma treatment using a luminescence-based assay according to the manufacturer's instructions (GSH/GSSG-Glo, Promega). Briefly, cells were lysed in either total glutathione lysis reagent for total glutathione measurement or oxidized glutathione lysis reagent for GSSG measurement. Luciferin was added to all wells, followed by luciferin detection reagent. Luminescence was measured in Tecan multimode plate reader, and GSH/GSSG ratios were calculated after interpolation of glutathione concentrations from standard curves. GSHtracer (Ratiometric GSH probe; Tocris GmbH) was used to quantify total GSH levels by live-cell imaging. After treatment, cells were loaded with 5 μM of GSHtracer and incubated for 90 min at 37 °C. Cells were washed once in media and imaged with a 20x objective using a live cell high throughput imaging system (Operetta CLS; PerkinElmer). Algorithm-based quantitative image analysis was performed using dedicated software (Harmony 4.8; PerkinElmer). The ratio of fluorescence at F_510_/F_580_ correlates with GSH concentration.

### 2.6 Small interfering RNA-mediated knockdown of xCT

MeWo cells (1 × 10^4^) were seeded in 96-well plates. esiRNA targeted against multiple regions of human SLC7A11 mRNA (Sigma-Aldrich) or non-targeting control esiRNA (Luc) was transfected using *X-tremeGENE* siRNA reagent (Sigma-Aldrich) according to the manufacturer's recommendation. Twenty-four hours later, immunofluorescence staining was performed using a primary anti xCT antibody (Abcam) and a secondary antibody conjugated with the fluorophore Alexa Fluor 546 (Thermo Scientific). High content imaging was done as described above. Quantitative image analysis was performed to determine absolute signal levels from individually segmented cells. Alternatively, the xCT knockdown cells were plasma-treated for 60 s, and metabolic activity was measured after 24 h as described above. The xCT inhibitor sulfasalazine (SFL) and the γ-GCS inhibitor butathione sulfoximine (BSO) were obtained from Sigma-Aldrich.

### 2.7 Cutaneous melanoma biopsies and tissue sections

Metastatic lesions from five patients suffering from malignant melanoma stage IV (female: 1/male: 4; mean age 59) were surgically removed, and punch biopsies (diameter ~ 3 mm) were generated *ex vivo*. The study was approved by the Institutional Review Board of the University of Duisburg-Essen (ethical committee of the University of Duisburg-Essen) under the Institutional Review Board protocol no. 12-4961-BO. The punch biopsies of the patient samples were treated with the kINPen for 120s. The samples were then incubated in a 96 well plate containing fully supplemented RPMI for 24 h. Tissues were embedded in OCT (VWR) in disposable base molds (Thermo Fisher), snap-frozen in liquid nitrogen, and stored at −80 °C. Using a cryo-microtome (Leica Microsystems), samples were sectioned vertically (thickness: 6 μm) and placed on glass microscope slides (Thermo Fisher). After DAKO (Dako) block, cells were stained with DAPI to segment nuclei (Vecta shield) as well as anti-S100 (Santa Cruz), anti-xCT (Abcam), and anti-cleaved Caspase 3 (Cell Signaling) antibodies. Secondary antibodies conjugated with Alexa Fluor 546 or Alexa Fluor 647 (Thermo Scientific) were used. Slides were mounted using Vectashield (Biozol). Tissue sections were imaged using the high content imaging device Operetta CLS. Data acquisition and analyses were performed with the respective software of the device (Harmony 4.8; PerkinElmer).

### 2.8 Statistical analysis

All experiments were performed in at least three independent biological replicates. Bar graphs depict mean + SE (standard error). Two groups were compared using Student's t-test. One-way analysis of variance (ANOVA) with Dunnett's multiple comparison test was used when comparing more than two groups to the respective control group. Pearsons correlation coefficients were computed with a 95% confidence interval. *p*-values < 0.05 were considered statistically significant and labeled with a * within graphs as described in legends. Graphing and statistical analysis were performed using prism 8.3 (GraphPad software).

## 3 Results

### 3.1 A cell-based screen identified tumor cell lines ‘resistant’ to cold physical plasma

**Fig. 1:**
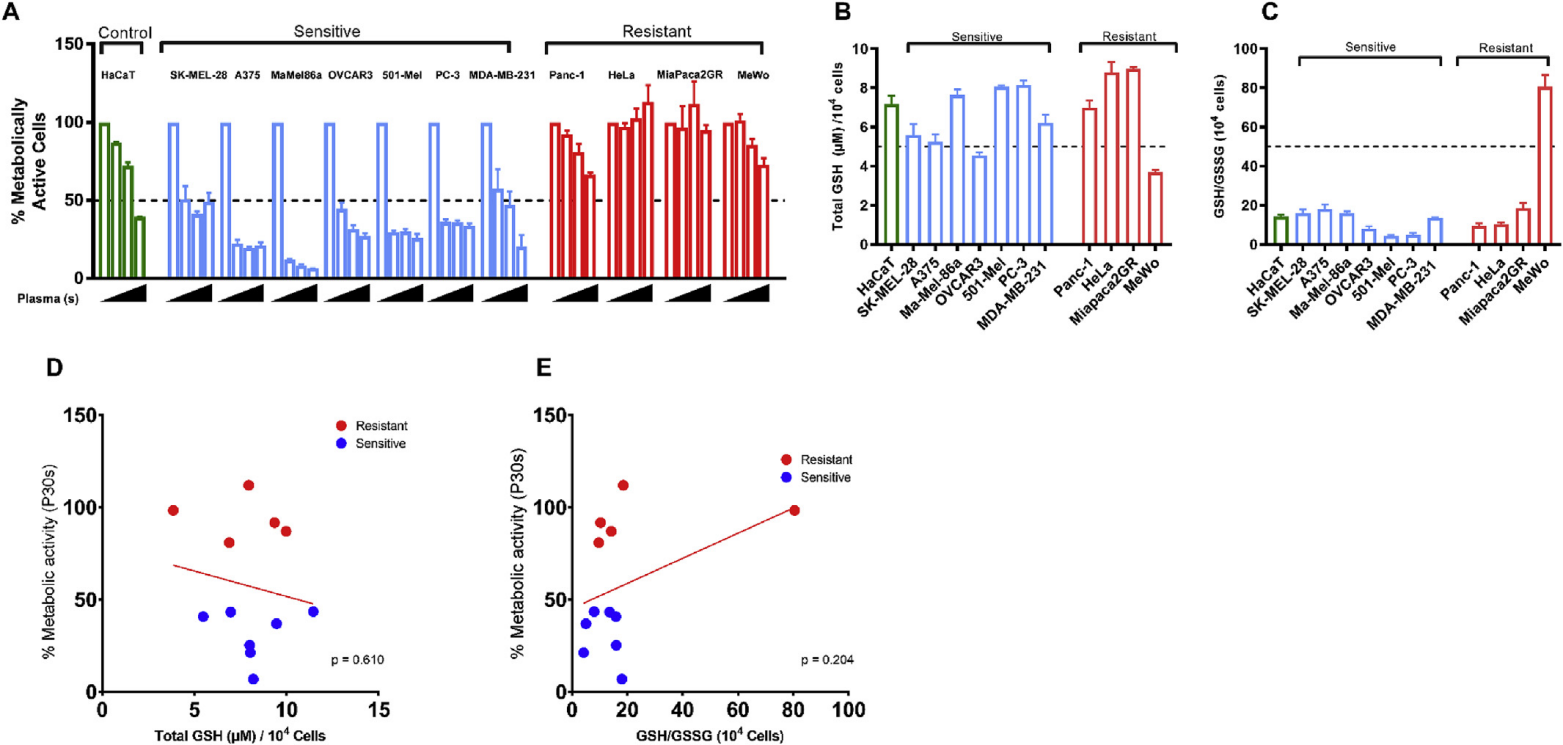
***Plasma treatment yielded a differential cytotoxic response in tumor cell lines in vitro.*** (A) Metabolic activity at 24 h of eleven different tumor cell lines treated with increasing doses of cold physical plasma (P30s, P60s, and P120s). For each cell line, the first bar indicates untreated cells to which the metabolic activity of plasma-treated cells was normalized (100%). Cell lines that showed >50% reduction in metabolic activity at P30s were categorized as ‘sensitive’, and <50% reduction was categorized as ‘resistant’ cell lines. (B) Basal glutathione (GSH) levels and (C) redox status expressed as GSH:GSSG ratio in cell lines included in the study. (D) Correlation analysis between total GSH and percent survival at P30s and (E) redox status and percent survival at P30s. The results are derived from three independent biological replicates and are shown as mean ± SEM.

To test the cytotoxicity of cold physical plasma, 11 different tumor cell lines were exposed to increasing plasma treatment times (30s, 60s, 120 s) and cultured for 24 h thereafter. The immortalized, non-malignant keratinocyte cell line HaCaT served as a control cell line. With 30s plasma treatment, an approximately 50% reduction in metabolic activity was observed in SK-MEL-28, A375, MaMel86a, OVCAR3, 501-Mel, PC-3, and MDA-MB-231 cells. These cell lines were categorized as ‘sensitive’. On the other hand, Panc-1, HeLa, Miapaca2GR, and MeWo were less affected by plasma treatment and hence termed ‘resistant’ (Fig. 1A). We further confirmed the cytotoxicity of plasma in these cell lines at 24 h by live-cell imaging of cytosolic signals (digital phase contrast) and a nuclear stain (sytox green) entering only cells with compromised membranes (Fig. S1). Then, the total GSH levels and GSH/GSSG ratio in the ‘sensitive’ and ‘resistant’ cell lines were quantified (Fig. 1B–C). There was no significant difference in the basal GSH or the GSH/GSSH ratio between the ‘sensitive’ and ‘resistant’ cell lines. Correlation analysis was performed on the percentage of metabolic activity of cells following 30 s plasma treatment against the total GSH content (Fig. 1D) or GSH/GSSG ratio (Fig. 1E). No correlation was observed. Hence, basal GSH levels did not predict the amplitude of cytotoxicity of cold plasma treatment in tumor cell lines.

### 3.2 S-glutathionylation and epigenetic inhibitors did not sensitize tumor cells to cold plasma

**Fig. 2:**
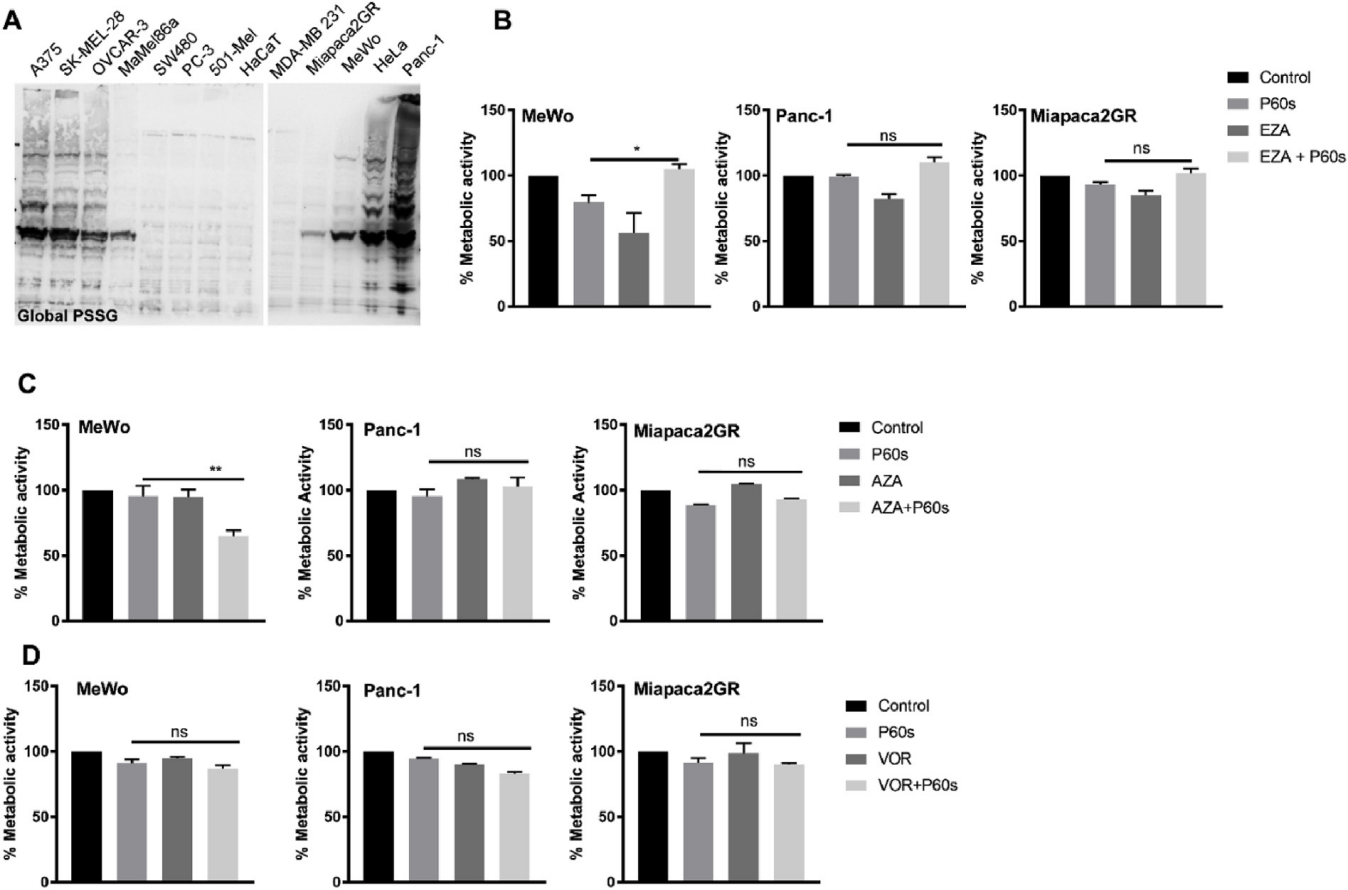
***S-glutathionylation, DNA methylation, and histone deacetylation inhibitors did not sensitize ‘resistant’ cell lines to cold physical plasma.*** (A) Global protein s-glutathionylation (PSSG) in tumor cell lysates. (B) Metabolic activity of ‘resistant’ cell lines upon pretreatment with GSTP1 inhibitor ezatiostat (EZA; 25 μM). (C) Metabolic activity of ‘resistant’ cell lines upon pretreatment with DNA methylation inhibitor azacytidine (AZA; 5 μM). (D) Metabolic activity of ‘resistant’ cell lines upon pretreatment HDAC inhibitor vorinostat (VOR; 10 μM). P30s and P60s are 30s and 60s of plasma treatment time, respectively. Data are mean ± SEM from three independent experiments. Statistical analysis was performed using Student's t-test with Welch's correction (B–D). ∗∗ = p < 0.01; ∗∗∗ = p < 0.001; ns = not significant.

S-glutathionylation is the most common post-translational modification in proteins at conserved cysteine residues leading to gain/loss of function of proteins. We hypothesized that s-glutathionylation could protect the tumor cells from oxidant-induced cell death. We assessed the global s-glutathionylation in tumor cell lysates by immunoblotting under non-reducing conditions using an anti-GSH antibody. Results indicated a different s-glutathionylation signature across the tumor cell lines investigated, with the Panc-1 cell line having extensively s-glutathionylated proteins (Fig. 2A). However, there was no definite correlation between the ‘resistant’ and ‘sensitive’ cell lines indicating that s-glutathionylation was not a predictor of cold plasma-induced toxicity. To validate this finding, we pretreated the tumor cells with 25 μM of ezatiostat (EZA), a known inhibitor of glutathione s-transferase 1 (GSTP1) followed by cold plasma treatment. The combination treatment did not sensitize the ‘resistant’ or ‘sensitive’ tumor cell lines to cold plasma (EZA vs. EZA + P60s), indicating that GSTP1 mediated s-glutathionylation did not influence the cytotoxic response to cold plasma treatment in tumor cells (Figs. 2B and S3). Alternatively, epigenetic alterations have been previously implicated in chemoresistance. Hence, we pretreated the cell lines with the DNA methylation inhibitor azacytidine (AZA, 5 μM) or the histone deacetylase inhibitor vorinostat (VOR, 10 μM) followed by cold plasma treatment. Azacytidine showed a modest additive effect in MeWo cells in combination with cold plasma (Fig. 2C), whereas other cell lines remained unaltered in their metabolic activity. However, vorinostat did not have any effect in combination with cold plasma (Fig. 2D). These experiments were also carried out in representative ‘sensitive’ cell lines (SK-MEL-28 and MaMel86a), and the combination of azacytidine with cold plasma (P30s) did not lead to an additive reduction in metabolic activity in MaMel86a and SK-MEL-28 cell lines (Fig. S2A). However, vorinostat showed an additive effect upon the combination with cold plasma in SK-MEL-28 cells (Fig. S2).

### 3.3 Total and reduced glutathione levels content were maintained in cells ‘resistant'to cold plasma

**Fig. 3:**
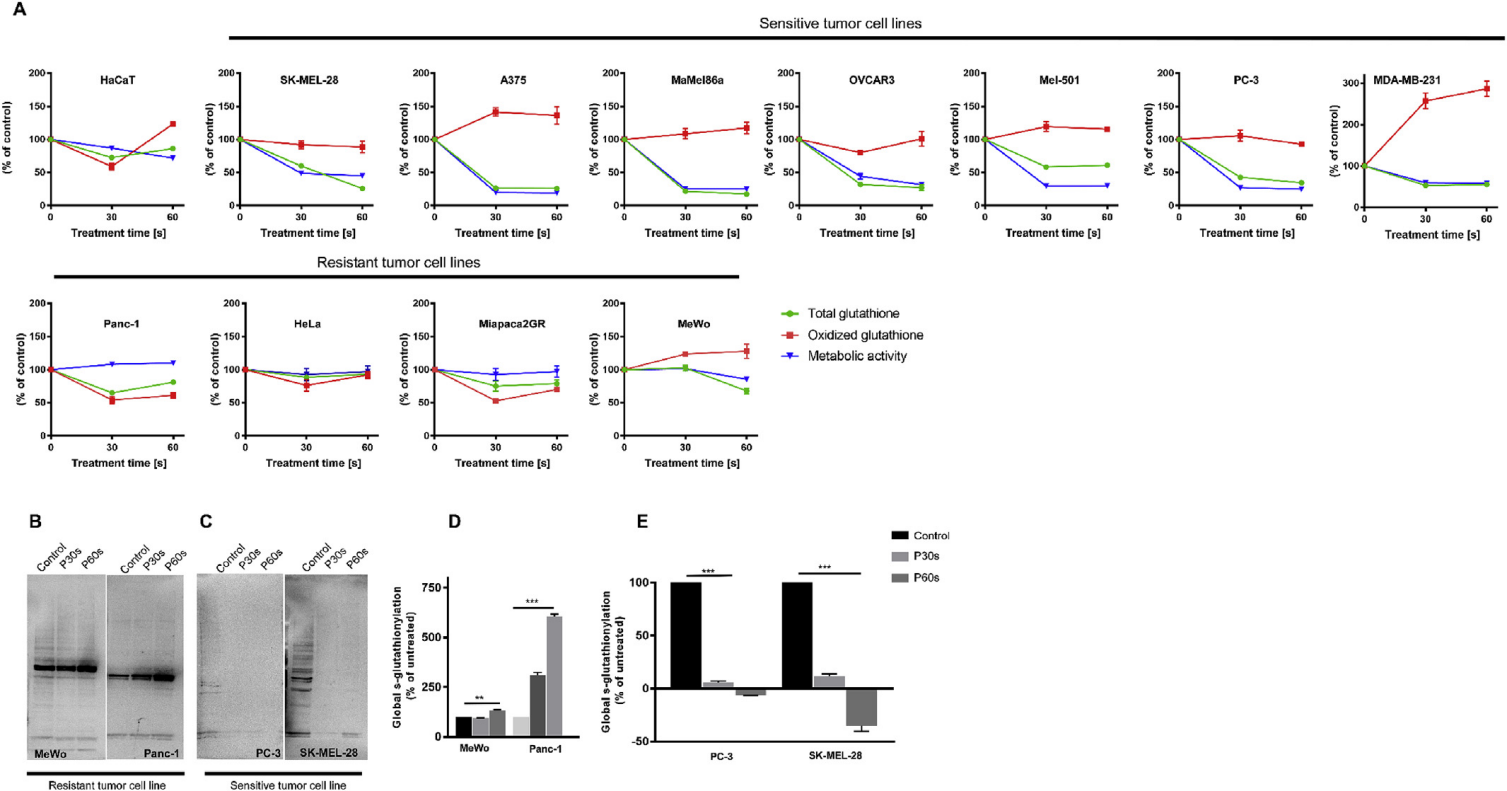
***Altered GSH homeostasis in ‘resistant’ tumor cell lines.*** (A) Graphical representation of metabolic activity, total GSH, and oxidized GSH at 6 h in ‘sensitive’ and ‘resistant’ cell lines post plasma treatment. (B–E) Immunoblotting and quantification of global s-glutathionylation under non-reducing conditions in ‘sensitive’ (PC-3, SK-MEL-28) and ‘resistant’ (MeWo and Panc-1) cell lines following cold physical plasma treatment. P30s and P60s are 30s and 60s of plasma treatment time, respectively. Data are mean ± SEM from three independent experiments. Statistical analysis was performed using Student's t-test with Welch's correction (D and E). ∗∗ = p < 0.01; ∗∗∗ = p < 0.001.

Total and oxidized glutathione (GSH) was measured at 6 h in tumors cell lines following exposure to 30 s and 60 s plasma treatment. This was correlated with metabolic activity, as previously determined. Total GSH levels were consistently depleted in cold plasma ‘sensitive’ tumor cell lines; however, the GSH levels were in a steady-state in the ‘resistant’ tumor cell lines. Furthermore, there was a concordant increase in oxidized GSH in ‘sensitive’ cells but not in ‘resistant’ cells. These observations suggested that there is differential regulation of GSH in ‘sensitive’ compared to ‘resistant’ tumor cell lines (Fig. 3A). To validate this, representative ‘resistant’ cell lines (MeWo and Panc-1) and ‘sensitive’ cell lines (PC-3 and SK-MEL-28) were quantified following plasma treatment by immunoblotting for global s-glutathionylation using an anti-GSH antibody (Fig. 3B–E) or a fluorescent GSH-tracer dye (Fig. S3). There was an increase of total GSH and global s-glutathionylation levels in ‘resistant’ cells and a decrease in ‘sensitive’ cells, which indicated an altered GSH homeostasis following plasma treatment.

### 3.4 xCT expression correlated to the ‘resistance’ to cold plasma treatment

**Fig. 4:**
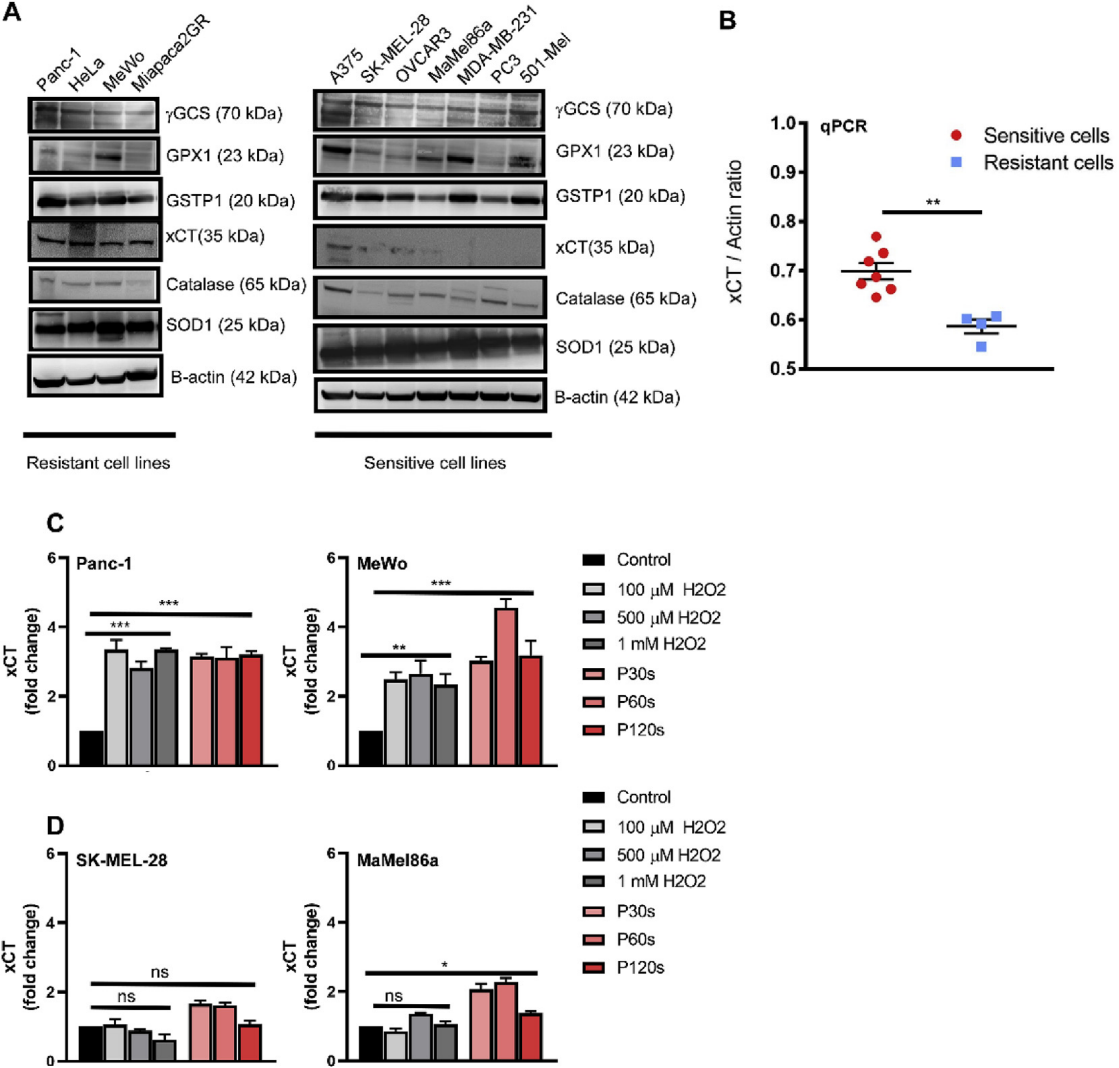
***xCT was preferentially expressed in ‘resistant’ tumor cells.*** (A) Immunoblotting analysis of enzymes involved in GSH homeostasis (γ-GCS, GPX1, GSTP1, xCT) and antioxidant enzymes (SOD1, catalase) with β-actin as the loading control. (B) Confirmation of increased basal expression of xCT in ‘resistant’ cell lines by qPCR analysis. (C–D) Fold change in xCT mRNA expression following treating the ‘resistant’ (C) and ‘sensitive’ cells (D) with cold plasma or hydrogen peroxide (H_2_O_2_). P30s, P60s, and P120 are 30s, 60s, and 120s of plasma treatment time, respectively. Data are mean ± SEM from three independent experiments. Statistical analysis was performed using Student's t-test with Welch's correction (B) and one-way anova with Dunnett's post-test for multiple comparisons (C–D). ∗ = p < 0.05; ∗∗ = p < 0.01; ∗∗∗ = p < 0.001; ns = not significant.

In order to identify the pathways responsible for ‘resistance’ towards cold plasma, the levels of proteins involved in the GSH biosynthesis (γ-GCS, GPX1, GSTP1, xCT) and antioxidant enzymes (SOD1 and catalase) were investigated by immunoblotting. Inconclusive protein levels of γ-GCS, GPX1, GSTP1, SOD1, and catalase were observed. Remarkably, xCT protein expression was high in the ‘resistant’ cell lines, whereas it was barely detectable in ‘sensitive’ cell lines (Fig. 4A). This expression pattern was confirmed at the mRNA level by qPCR in both groups (Fig. 4B). Furthermore, treating the ‘resistant’ cells (Panc-1 and MeWo) with cold plasma or hydrogen peroxide (H_2_O_2_) did induce high and stable expression of xCT mRNA 6 h later, suggesting that its expression was dependent on ROS-induced signaling (Fig. 4C). However, this stable expression was not seen in ‘sensitive’ cell lines SK-MEL 28 and MaMel86a (Fig. 4D).

### 3.5 Inhibition of xCT restored the sensitivity of cold plasma in ‘resistant’ cell lines

**Fig. 5:**
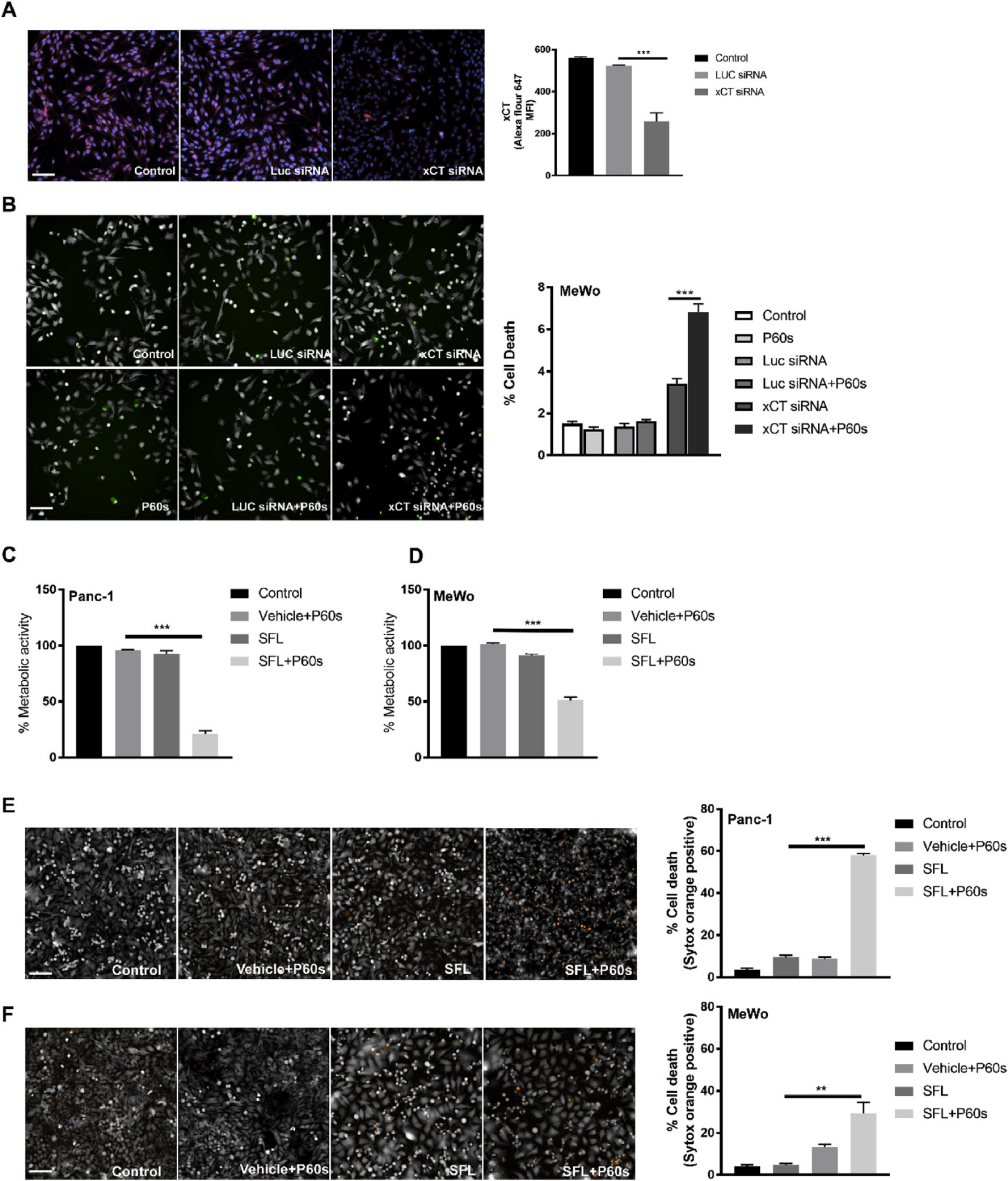
***Inhibition of xCT sensitized ‘resistant’ tumor cells to cold plasma.*** (A) Immunofluorescence and quantification of xCT expression (red) and nuclei (blue) in MeWo cells following esiRNA mediated knockdown of xCT after 24 h. (B) Cytotoxicity in MeWo cells following xCT knockdown and 60 s of cold plasma treatment (P60s). (C–D) Metabolic activity of ‘resistant’ cell lines (Panc-1 and MeWo) pretreated with sulfasalazine (SFL, 500 μM; 24 h) followed by exposure to 60 s of plasma treatment. (E–F) ‘resistant’ cell lines (Panc-1 and MeWo) were treated with 500 μM of SFL for 24 h, followed by exposure to cold physical plasma and further incubation for 24 h before staining with sytox orange to determine the percent of dead cells. Results are shown as mean + SEM. Statistical analysis was performed by student's t-test with Welch's correction. ∗∗ = p < 0.01; ∗∗∗ = p < 0.001. Scale bar: 50 μm. (For interpretation of the references to color in this figure legend, the reader is referred to the Web version of this article.)

To test whether xCT ablation sensitized the tumor cells to plasma-induced cytotoxic effects, we performed knockdown experiments using esiRNA against xCT mRNA. Knockdown efficiency was determined in the MeWo cell line after 24 h (Fig. 5A). These cells were then treated with cold plasma for 60 s and incubated for an additional 24 h. Sytox green viability staining showed increased cytotoxicity upon xCT knockdown, which was significantly increased upon treatment with cold plasma (Fig. 5B). To tested whether pharmacological inhibition of xCT sensitized the ‘resistant’ tumor cell lines to cold plasma, we pretreated Panc-1 and MeWo cells with sulfasalazine (SFL, 500 μM) for 16 h and then exposed the cells to cold plasma. Pretreatment sensitized the ‘resistant’ cells by significantly decreasing their metabolic activity upon plasma treatment (Fig. 5C–D). Furthermore, the viability of these cells was determined by sytox orange staining, and a significant increase of cytotoxicity following xCT inhibition and exposure to plasma was observed (Fig. 5E–F). Similar results were obtained by inhibiting the enzyme critical for GSH biosynthesis, γ-GCS, using butathione sulfoximine (BSO) (Fig. S4). These results indicated that xCT plays a significant role in tumor cell survival and conferring ‘resistance’ to cold plasma treatment.

### 3.6 xCT expression correlates to cold plasma sensitivity in human melanoma samples *ex vivo*

**Fig. 6:**
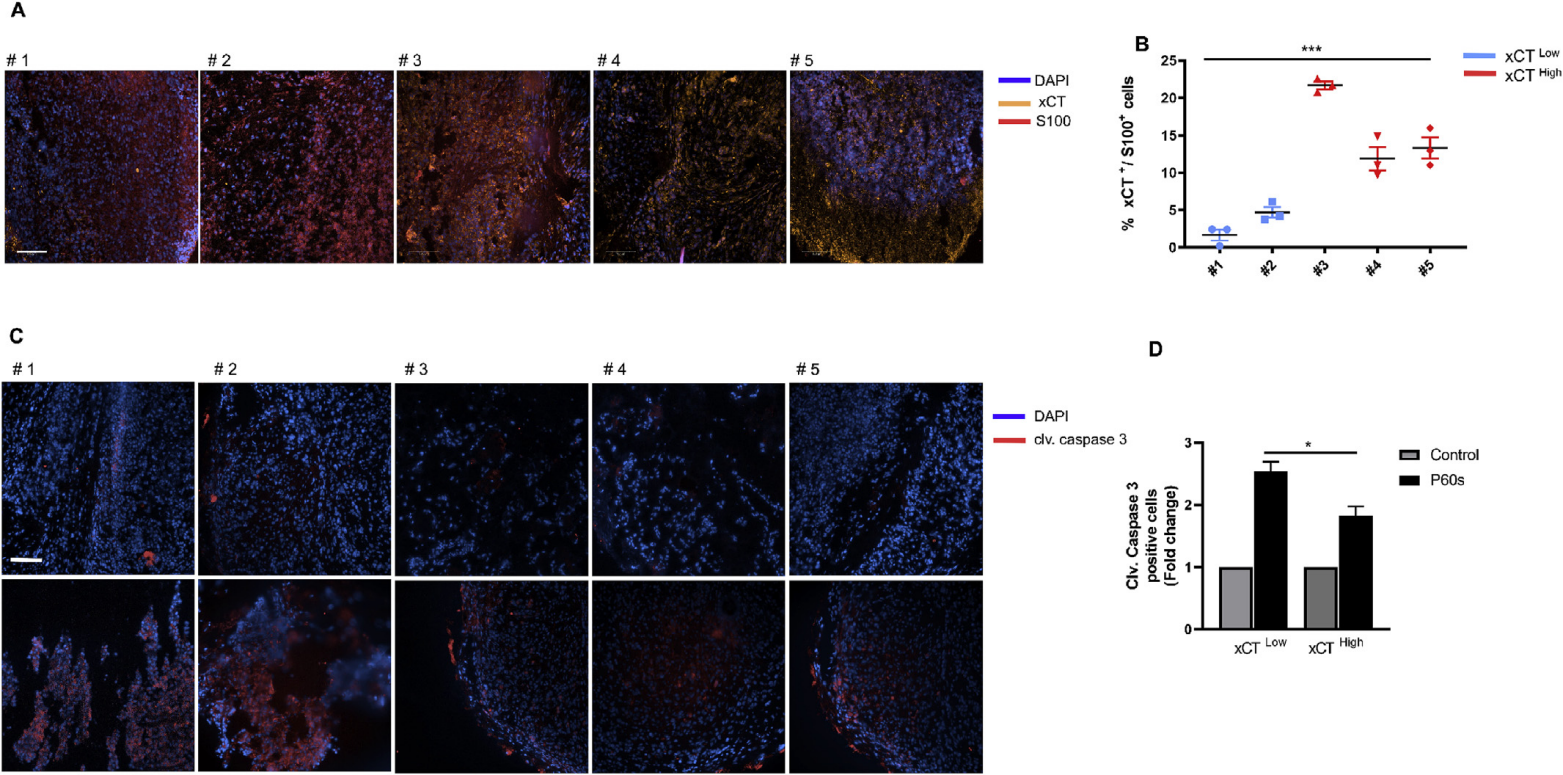
***xCT was differentially expressed in human melanoma patients*** (A) Representative images of immunostaining staining of malignant metastatic melanoma samples from five patients using antibodies against melanoma tumor antigen S100 (Red) and xCT (yellow). DAPI (blue) served as the nuclear counterstain. (B) Quantification of xCT expression from (A) expressed as % xCT/S100 double-positive cells. Statistical analysis was performed by one way anova, ***p < 0.001. (C) Immunostaining of cleaved caspase 3 (red) following *ex vivo* treatment of human melanoma punch biopsies with cold physical plasma for 60s (P60s). DAPI (blue) served as the nuclear counterstain. (D) Quantification of cleaved caspase 3 intensity from (C) expressed as mean fluorescent intensity from 1000 DAPI positive cells. Statistical analysis was performed by student's t-test with Welch's correction, *p < 0.05. Scale bar: 250 μm. (For interpretation of the references to color in this figure legend, the reader is referred to the Web version of this article.)

To validate whether xCT is expressed in primary human tumor tissue, malignant melanoma tissue samples were analyzed. Immunohistochemical analysis for the melanoma antigen S100 and xCT were performed. Quantification of xCT in S100-positive tumor cells showed a high degree of inter-patient heterogeneity. Two out of the five samples had an xCT staining score of <10%, whereas the remaining three had a score of >10% (Fig. 6A–B). The punch biopsies of the corresponding patient samples were then treated with cold physical plasma and analyzed for caspase 3 activity. Plasma treatment increased cleaved caspase 3 levels in samples that had lower xCT expression in comparison with samples with higher xCT expression (Fig. 6 C-D).

## 4 Discussion

Cold physical plasma is an emerging therapeutic tool against cancer. This involves direct application of cold plasma-derived short-lived and long-lived RONS [25,26] or indirect application using cold plasma-activated liquids [[27], [28], [29]] that specifically target tumor cells with altered redox balance. Several studies have demonstrated that cold plasma-derived RONS kill tumor cell lines but spare healthy cells *in vitro* [[30], [31], [32]]. However, we observed discrepancies in the sensitivity of certain tumor cell lines to cold plasma. Our previous unpublished observations revealed that melanoma cell line MeWo was ‘resistant’ to cold physical plasma when compared with SK-MEL-28. In the current study, we identified four tumor cell lines ‘resistant’ to cold plasma and investigated the pathways that may contribute to such ‘resistant’ phenotype.

Epigenetic changes in tumors are associated with tumor progression by altering the expression of genes involved in DNA repair, drug uptake, cell cycle checkpoints, and apoptosis [33]. Regions of high CpG dinucleotide density (CpG islands) located in the promoters of tumor suppressor and housekeeping genes are usually free of methylation. In cancer cells, the CpG islands become hypermethylated, contributing to the silencing of tumor suppressor genes [34]. The acetylation of histones makes DNA more accessible to transcription factors leading to enhanced gene expression. However, tumor cells have enhanced histone deacetylase activity that leads to condensation of chromatin resulting in the inhibition of expression of several genes [35]. Here, aberrant epigenetic mechanisms mediated by DNA methylation (CpG islands) and histone acetylation play a crucial role in drug resistance [36]. Previous studies have demonstrated that the DNA hypomethylating agents azacytidine and the histone deacetylase inhibitor vorinostat were more effective in reactivating transcription of epigenetically silenced genes leading to sensitization to chemotherapy and radiotherapy [37,38]. On similar lines, our results indicate that both these two epigenetic reversing drugs did not sensitize the ‘resistant’ tumor cell lines to cold plasma, suggesting other pathways enabling the ‘resistant’ phenotype. However, as an exception, we observed additive cytotoxicity in the MeWo cell line using azacytidine, implying that DNA methylation could play a role in ‘resistance’ towards plasma treatment in these cells. On the other hand, treating the ‘sensitive’ cells with these DNA modifying drugs did not induce additive toxicity in combination with cold plasma with the exception of SK-MEL-28 cells upon treatment with vorinostat.

Glutathione (GSH) is a tripeptide in its reduced form and the most abundant non-protein thiol in mammalian cells. It acts as a reducing agent and is a significant antioxidant governing the redox status of the cell. Upon oxidative stress, GSH is oxidized to GSSG by RONS, leading to a reduction of intracellular GSH levels [39]. This depletion of GSH is shown to potentiate drug-mediated cell death in tumor cells [40,41]. Since cold plasma is a pro-oxidant therapy, GSH seemed to be an ideal marker predicting treatment outcome. However, our findings did not establish any correlation between basal GSH levels and increased sensitivity to cold plasma-induced toxicity. The observed heterogeneous levels of GSH could be attributed to the complex regulation of GSH biosynthesis across the cell lines.

Protein s-glutathionylation (P-SSG) is a reversible oxidative posttranslational modification at conserved cysteine residues in proteins. S-glutathionylation often leads to a conformational change in protein structure with subsequent gain or loss of function, resulting in alternative signaling patterns. The unique feature of s-glutathionylation is its involvement in both cell survival and cell death mechanisms [[42], [43], [44]]. S-glutathionylation of the 20S proteasome subunit raises the degradation of oxidized proteins, thereby increasing cell survival during stress conditions [45]. Furthermore, consensus exists that protein s-glutathionylation preserves proteins in functionally silent states from irreversible over-oxidation [46,47]. Our observations indicate that there was no significant difference in global s-glutathionylation among ‘sensitive’ and ‘resistant’ cell lines at basal conditions. However, upon plasma treatment, we could see a decrease in GSH and s-glutathionylation levels in ‘sensitive’ cell lines, and steady GSH levels and an increase in s-glutathionylation in ‘resistant’ cell lines. There was significant s-glutathionylation in ‘resistant’ cell lines and the GSTP1 inhibitor ezatiostat did not sensitize the ‘resistant’ cell lines to cold plasma, suggesting an altered GSH metabolism in response to plasma-derived RONS in ‘resistant’ cell lines.

GSH biosynthesis involves xCT coded by the SLC7A11 gene, a plasma membrane-bound sodium-independent antiporter that imports cystine and exports glutamate in a 1:1 ratio [48]. Intracellular cystine is reduced to cysteine and serves as a limiting precursor of GSH synthesis by γ-glutamylcysteine synthetase (γ-GCS) [39]. The expression of xCT is deregulated in multiple cancers. Its overexpression has been associated with poor prognosis [[49], [50], [51]]. Another study had described the induction of xCT expression upon oxidative stress [52]. In our study, xCT appeared to be differentially expressed between the ‘sensitive’ and ‘resistant’ tumor cell lines. Furthermore, we demonstrated that cold plasma or H_2_O_2_ treatment induces the expression of xCT in ‘resistant’ cell lines but not in ‘sensitive’ cell lines. This implies that ‘resistant’ cell lines have constitutive access of cystine for GSH biosynthesis at basal and oxidative stress conditions. The expression of SLC7A11 is regulated by NRF2 and ATF4 transcription factors upon multiple cellular stress conditions [[53], [54], [55]]. It could be speculated that the ‘sensitive’ cell lines might have impaired NRF2/ATF4 signaling, which may be responsible for increased cytotoxicity observed following cold plasma treatment.

Previous studies demonstrated that xCT inhibition reduced tumor cell invasion and survival *in vitro* [50,56]. In our study, the esiRNA-mediated knockdown of xCT also increased the basal levels of cell death in MeWo cells and demonstrated additive toxicity upon plasma treatment. Furthermore, pharmacological inhibition of xCT by sulfasalazine confirmed this claim that xCT expression determines the sensitivity of the tumor cells to cold plasma. Recent studies have also shown that inhibition of xCT improves the radiosensitivity of tumor cells via glutathione reduction [57,58]. In several reports, depleted GSH in tumor cells by inhibiting γ-GCS was performed, ultimately leading to tumor cell sensitization to chemotherapies [59,60]. In our work, we inhibited γ-GCS (downstream of xCT) by BSO, which sensitized ‘resistant’ cell lines to cold plasma treatment via depletion of GSH. This indicated that GSH homeostasis is critical for cell survival following cold plasma treatment. However, sensitization potency was 2-fold higher upon inhibition of xCT in comparison with γ-GCS, and the mRNA expression of xCT had more predictive power for chemoresistance than that of γ-GCS and GSTP1 [61]. Taken together, our findings implicate xCT expression predicting the sensitivity of tumor cells to cold plasma.

The development of resistance to treatment in cancer patients remains a major clinical issue. Melanoma is one of the tumors with high mutational frequency [62] and develops resistance to many therapies [63], hence serving as an ideal model for testing novel therapies. Erastin and sulfasalazine have been shown to inhibit xCT activity *in vitro* and *in vivo* in multiple tumor models [49,51,57]. Furthermore, xCT knockout mice were viable and fertile without any loss in T-cell function or antitumor immune response [64]. Our previous findings suggest that the combination of cold plasma and anti-cancer agents can evoke a strong synergistic anti-tumor response [21,30]. Potentially, xCT inhibitors may sensitize malignant melanoma to cold plasma treatment in a combination approach.

Similar to head and neck cancer therapy [[65], [66], [67]], the antitumor efficacy of cold physical plasma in patients with melanoma could be carried out in a clinical setting in the future. Our immunohistochemical analysis of melanoma biopsies revealed a heterogeneous expression of xCT in tumor tissue. However, increased active caspase-3 in punch biopsies of melanoma patients following *ex vivo* plasma treatment confirms our hypothesis that xCT expression correlates to the tumor cells’ sensitivity towards cold plasma treatment. Since xCT determines the sensitivity of tumor cells to cold plasma treatment *in vitro* and *ex vivo*, its expression in melanoma patients could serve as a predictive marker for the efficacy cold plasma therapy, provided such concept would be validated further.

## Declaration of competing interest

The authors have no conflict of interest to declare.

## References

[1] Butcher L. (2008). Solid tumors: prevalence, economics, and implications for payers and purchasers. Biotechnol. Healthc..

[2] von Woedtke T., Reuter S., Masur K., Weltmann K.D. (2013). Plasmas for medicine. Phys. Rep..

[3] Gu S., Chen C., Jiang X., Zhang Z. (2016). ROS-mediated endoplasmic reticulum stress and mitochondrial dysfunction underlie apoptosis induced by resveratrol and arsenic trioxide in A549 cells. Chem. Biol. Interact..

[4] Suzuki-Karasaki Y. (2016). Tumor-targeting killing of multidrug-resistant human aggressive cancer cells by plasma-activated media via mitochondrial and endoplasmic reticulum damages. Int. J. Mol. Med..

[5] Adachi T., Tanaka H., Nonomura S., Hara H., Kondo S., Hori M. (2015). Plasma-activated medium induces A549 cell injury via a spiral apoptotic cascade involving the mitochondrial-nuclear network. Free Radic. Biol. Med..

[6] Bekeschus S., Wende K., Hefny M.M., Rodder K., Jablonowski H., Schmidt A., Woedtke T.V., Weltmann K.D., Benedikt J. (2017). Oxygen atoms are critical in rendering THP-1 leukaemia cells susceptible to cold physical plasma-induced apoptosis. Sci. Rep..

[7] Wende K., Williams P., Dalluge J., Gaens W.V., Aboubakr H., Bischof J., von Woedtke T., Goyal S.M., Weltmann K.D., Bogaerts A. (2015). Identification of the biologically active liquid chemistry induced by a nonthermal atmospheric pressure plasma jet. Biointerphases.

[8] Bekeschus S., Mueller A., Miller V., Gaipl U., Weltmann K.-D. (2018). Physical plasma elicits immunogenic cancer cell death and mitochondrial singlet oxygen. IEEE Trans. Radiat. Plasma Med. Sci..

[9] Bekeschus S., Lin A., Fridman A., Wende K., Weltmann K.D., Miller V. (2018). A comparison of floating-electrode DBD and kINPen jet: plasma parameters to achieve similar growth reduction in colon cancer cells under standardized conditions. Plasma Chem. Plasma Process..

[10] Schmidt A., Bekeschus S., Jablonowski H., Barton A., Weltmann K.D., Wende K. (2017). Role of ambient gas composition on cold physical plasma-elicited cell signaling in keratinocytes. Biophys. J..

[11] Bekeschus S., Schmidt A., Niessner F., Gerling T., Weltmann K.D., Wende K. (2017). Basic research in plasma medicine - a throughput approach from liquids to cells. J. Vis. Exp..

[12] Bauer G. (2019). The synergistic effect between hydrogen peroxide and nitrite, two long-lived molecular species from cold atmospheric plasma, triggers tumor cells to induce their own cell death. Redox Biol..

[13] Bekeschus S., Kolata J., Winterbourn C., Kramer A., Turner R., Weltmann K.D., Broker B., Masur K. (2014). Hydrogen peroxide: a central player in physical plasma-induced oxidative stress in human blood cells. Free Radic. Res..

[14] Girard P.M., Arbabian A., Fleury M., Bauville G., Puech V., Dutreix M., Sousa J.S. (2016). Synergistic effect of H2O2 and NO2 in cell death induced by cold atmospheric He plasma. Sci. Rep..

[15] Balzer J., Demir E., Kogelheide F., Fuchs P.C., Stapelmann K., Opländer C. (2019). Cold atmospheric plasma (CAP) differently affects migration and differentiation of keratinocytes via hydrogen peroxide and nitric oxide-related products. Clin. Plas. Med..

[16] Shekhter A.B., Pekshev A.V., Vagapov A.B., Telpukhov V.I., Panyushkin P.V., Rudenko T.G., Fayzullin A.L., Sharapov N.A., Vanin A.F. (2018). Physicochemical parameters of NO-containing gas flow affect wound healing therapy. An experimental study. Eur. J. Pharm. Sci..

[17] Suschek C.V., Opländer C. (2016). The application of cold atmospheric plasma in medicine: the potential role of nitric oxide in plasma-induced effects. Clin. Plas. Med..

[18] Verlackt C.C.W., Neyts E.C., Jacob T., Fantauzzi D., Golkaram M., Shin Y.K., van Duin A.C.T., Bogaerts A. (2015). Atomic-scale insight into the interactions between hydroxyl radicals and DNA in solution using the ReaxFF reactive force field. New J. Phys..

[19] Priya Arjunan K., Morss Clyne A. (2011). Hydroxyl radical and hydrogen peroxide are primarily responsible for dielectric barrier discharge plasma-induced angiogenesis. Plasma Process. Polym..

[20] Uchiyama H., Zhao Q.L., Hassan M.A., Andocs G., Nojima N., Takeda K., Ishikawa K., Hori M., Kondo T. (2015). EPR-spin trapping and flow cytometric studies of free radicals generated using cold atmospheric argon plasma and X-ray irradiation in aqueous solutions and intracellular milieu. PLoS One.

[21] Sagwal S.K., Pasqual-Melo G., Bodnar Y., Gandhirajan R.K., Bekeschus S. (2018). Combination of chemotherapy and physical plasma elicits melanoma cell death via upregulation of SLC22A16. Cell Death Dis..

[22] Martincorena I., Campbell P.J. (2015). Somatic mutation in cancer and normal cells. Science.

[23] Batlle E., Clevers H. (2017). Cancer stem cells revisited. Nat. Med..

[24] Moulder S. (2010). Intrinsic resistance to chemotherapy in breast cancer. Women's Health.

[25] Liedtke K.R., Diedrich S., Pati O., Freund E., Flieger R., Heidecke C.D., Partecke L.I., Bekeschus S. (2018). Cold physical plasma selectively elicits apoptosis in murine pancreatic cancer cells in vitro and in ovo. Anticancer Res..

[26] Bekeschus S., Freund E., Spadola C., Privat-Maldonado A., Hackbarth C., Bogaerts A., Schmidt A., Wende K., Weltmann K.D., von Woedtke T. (2019). Risk assessment of kINPen plasma treatment of four human pancreatic cancer cell lines with respect to metastasis. Cancers.

[27] Freund E., Liedtke K.R., Gebbe R., Heidecke A.K., Partecke L.-I., Bekeschus S. (2019). In vitro anticancer efficacy of six different clinically approved types of liquids exposed to physical plasma. IEEE Trans. Rad. Plas. Med. Sci..

[28] Tanaka H., Nakamura K., Mizuno M., Ishikawa K., Takeda K., Kajiyama H., Utsumi F., Kikkawa F., Hori M. (2016). Non-thermal atmospheric pressure plasma activates lactate in Ringer's solution for anti-tumor effects. Sci. Rep..

[29] Freund E., Liedtke K.R., van der Linde J., Metelmann H.R., Heidecke C.D., Partecke L.I., Bekeschus S. (2019). Physical plasma-treated saline promotes an immunogenic phenotype in CT26 colon cancer cells in vitro and in vivo. Sci. Rep..

[30] Gandhirajan R.K., Rodder K., Bodnar Y., Pasqual-Melo G., Emmert S., Griguer C.E., Weltmann K.D., Bekeschus S. (2018). Cytochrome C oxidase inhibition and cold plasma-derived oxidants synergize in melanoma cell death induction. Sci. Rep..

[31] Hasse S., Seebauer C., Wende K., Schmidt A., Metelmann H.R., von Woedtke T., Bekeschus S. (2019). Cold argon plasma as adjuvant tumour therapy on progressive head and neck cancer: a preclinical study. Appl. Sci.-Basel.

[32] Pasqual-Melo G., Gandhirajan R.K., Stoffels I., Bekeschus S. (2018). Targeting malignant melanoma with physical plasmas. Clin. Plas. Med..

[33] Dawson M.A., Kouzarides T. (2012). Cancer epigenetics: from mechanism to therapy. Cell.

[34] Bennett R.L., Licht J.D. (2018). Targeting epigenetics in cancer. Annu. Rev. Pharmacol. Toxicol..

[35] Ellis L., Atadja P.W., Johnstone R.W. (2009). Epigenetics in cancer: targeting chromatin modifications. Mol. Cancer Ther..

[36] Montellier E., Gaucher J. (2019). Targeting the interplay between metabolism and epigenetics in cancer. Curr. Opin. Oncol..

[37] Plumb J.A., Strathdee G., Sludden J., Kaye S.B., Brown R. (2000). Reversal of drug resistance in human tumor xenografts by 2'-deoxy-5-azacytidine-induced demethylation of the hMLH1 gene promoter. Cancer Res..

[38] Cameron E.E., Bachman K.E., Myohanen S., Herman J.G., Baylin S.B. (1999). Synergy of demethylation and histone deacetylase inhibition in the re-expression of genes silenced in cancer. Nat. Genet..

[39] Forman H.J., Zhang H., Rinna A. (2009). Glutathione: overview of its protective roles, measurement, and biosynthesis. Mol. Asp. Med..

[40] Armstrong J.S., Steinauer K.K., Hornung B., Irish J.M., Lecane P., Birrell G.W., Peehl D.M., Knox S.J. (2002). Role of glutathione depletion and reactive oxygen species generation in apoptotic signaling in a human B lymphoma cell line. Cell Death Differ..

[41] Friesen C., Kiess Y., Debatin K.M. (2004). A critical role of glutathione in determining apoptosis sensitivity and resistance in leukemia cells. Cell Death Differ..

[42] Pastore A., Piemonte F. (2013). Protein glutathionylation in cardiovascular diseases. Int. J. Mol. Sci..

[43] Mieyal J.J., Gallogly M.M., Qanungo S., Sabens E.A., Shelton M.D. (2008). Molecular mechanisms and clinical implications of reversible protein S-glutathionylation. Antioxidants Redox Signal..

[44] Gandhirajan R.K., Jain M., Walla B., Johnsen M., Bartram M.P., Huynh Anh M., Rinschen M.M., Benzing T., Schermer B. (2016). Cysteine S-glutathionylation promotes stability and activation of the hippo downstream effector transcriptional Co-activator with PDZ-binding Motif (TAZ). J. Biol. Chem..

[45] Demasi M., Hand A., Ohara E., Oliveira C.L., Bicev R.N., Bertoncini C.A., Netto L.E. (2014). 20S proteasome activity is modified via S-glutathionylation based on intracellular redox status of the yeast Saccharomyces cerevisiae: implications for the degradation of oxidized proteins. Arch. Biochem. Biophys..

[46] Hill B.G., Bhatnagar A. (2012). Protein S-glutathiolation: redox-sensitive regulation of protein function. J. Mol. Cell. Cardiol..

[47] Boivin B., Yang M., Tonks N.K. (2010). Targeting the reversibly oxidized protein tyrosine phosphatase superfamily. Sci. Signal..

[48] Bannai S. (1986). Exchange of cystine and glutamate across plasma membrane of human fibroblasts. J. Biol. Chem..

[49] Guo W., Zhao Y., Zhang Z., Tan N., Zhao F., Ge C., Liang L., Jia D., Chen T., Yao M. (2011). Disruption of xCT inhibits cell growth via the ROS/autophagy pathway in hepatocellular carcinoma. Cancer Lett..

[50] Timmerman L.A., Holton T., Yuneva M., Louie R.J., Padro M., Daemen A., Hu M., Chan D.A., Ethier S.P., van 't Veer L.J. (2013). Glutamine sensitivity analysis identifies the xCT antiporter as a common triple-negative breast tumor therapeutic target. Cancer Cell.

[51] Ji X., Qian J., Rahman S.M.J., Siska P.J., Zou Y., Harris B.K., Hoeksema M.D., Trenary I.A., Heidi C., Eisenberg R. (2018). xCT (SLC7A11)-mediated metabolic reprogramming promotes non-small cell lung cancer progression. Oncogene.

[52] Lo M., Ling V., Wang Y.Z., Gout P.W. (2008). The xc- cystine/glutamate antiporter: a mediator of pancreatic cancer growth with a role in drug resistance. Br. J. Canc..

[53] Shih A.Y., Johnson D.A., Wong G., Kraft A.D., Jiang L., Erb H., Johnson J.A., Murphy T.H. (2003). Coordinate regulation of glutathione biosynthesis and release by Nrf2-expressing glia potently protects neurons from oxidative stress. J. Neurosci..

[54] Ye P., Mimura J., Okada T., Sato H., Liu T., Maruyama A., Ohyama C., Itoh K. (2014). Nrf2- and ATF4-dependent upregulation of xCT modulates the sensitivity of T24 bladder carcinoma cells to proteasome inhibition. Mol. Cell. Biol..

[55] Koppula P., Zhang Y., Shi J., Li W., Gan B. (2017). The glutamate/cystine antiporter SLC7A11/xCT enhances cancer cell dependency on glucose by exporting glutamate. J. Biol. Chem..

[56] Chen R.S., Song Y.M., Zhou Z.Y., Tong T., Li Y., Fu M., Guo X.L., Dong L.J., He X., Qiao H.X. (2009). Disruption of xCT inhibits cancer cell metastasis via the caveolin-1/beta-catenin pathway. Oncogene.

[57] Cobler L., Zhang H., Suri P., Park C., Timmerman L.A. (2018). xCT inhibition sensitizes tumors to gamma-radiation via glutathione reduction. Oncotarget.

[58] Nagane M., Kanai E., Shibata Y., Shimizu T., Yoshioka C., Maruo T., Yamashita T. (2018). Sulfasalazine, an inhibitor of the cystine-glutamate antiporter, reduces DNA damage repair and enhances radiosensitivity in murine B16F10 melanoma. PLoS One.

[59] Rocha C.R., Garcia C.C., Vieira D.B., Quinet A., de Andrade-Lima L.C., Munford V., Belizario J.E., Menck C.F. (2014). Glutathione depletion sensitizes cisplatin- and temozolomide-resistant glioma cells in vitro and in vivo. Cell Death Dis..

[60] Bansal A., Simon M.C. (2018). Glutathione metabolism in cancer progression and treatment resistance. J. Cell Biol..

[61] Dai Z., Huang Y., Sadee W., Blower P. (2007). Chemoinformatics analysis identifies cytotoxic compounds susceptible to chemoresistance mediated by glutathione and cystine/glutamate transport system xc. J. Med. Chem..

[62] Alexandrov L.B., Nik-Zainal S., Wedge D.C., Aparicio S.A., Behjati S., Biankin A.V., Bignell G.R., Bolli N., Borg A., Borresen-Dale A.L. (2013). Signatures of mutational processes in human cancer. Nature.

[63] Winder M., Viros A. (2018). Mechanisms of drug resistance in melanoma. Handb. Exp. Pharmacol..

[64] Arensman M.D., Yang X.S., Leahy D.M., Toral-Barza L., Mileski M., Rosfjord E.C., Wang F., Deng S., Myers J.S., Abraham R.T. (2019). Cystine-glutamate antiporter xCT deficiency suppresses tumor growth while preserving antitumor immunity. Proc. Natl. Acad. Sci. U. S. A..

[65] Metelmann H.-R., Seebauer C., Miller V., Fridman A., Bauer G., Graves D.B., Pouvesle J.-M., Rutkowski R., Schuster M., Bekeschus S. (2018). Clinical experience with cold plasma in the treatment of locally advanced head and neck cancer. Clin. Plas. Med..

[66] Schuster M., Rutkowski R., Hauschild A., Shojaei R.K., von Woedtke T., Rana A., Bauer G., Metelmann P., Seebauer C. (2018). Side effects in cold plasma treatment of advanced oral cancer—clinical data and biological interpretation. Clin. Plas. Med..

[67] Metelmann H.R., Seebauer C., Rutkowski R., Schuster M., Bekeschus S., Metelmann P. (2018). Treating cancer with cold physical plasma: on the way to evidence-based medicine. Contrib. Plasma Phys..

