# Supplementary material for: xCT (SLC7A11) expression confers intrinsic resistance to physical plasma treatment in tumor cells
Source: Redox Biol. 2020 Jan 3;30:101423. doi: 10.1016/j.redox.2019.101423 (PMC6957833; doi:10.1016/j.redox.2019.101423)
Supplement: Multimedia component 1 [file mmc1.docx]

**xCT (SLC7A11) expression confers intrinsic resistance to physical plasma treatment in tumor cells**

*Sander Bekeschus^1^, Sebastian Eisenmann^1^, Sanjeev Kumar Sagwal^1^, Gabriella Pasqual-Melo^1^ Juliane Moritz^1^, Yana Bodnar^1^, Juliane Moritz^1^, Broder Poschkamp^1,2^, Ingo Stoffels^2^, Steffen Emmert^3^, Thomas von Woedtke^1,5^, Madesh Muniswamy^6^, Klaus‑Dieter Weltmann^1^, *Rajesh Kumar Gandhirajan^1^

1. Leibniz Institute for Plasma Science and Technology (INP Greifswald), ZIK *plasmatis*, Felix‑Hausdorff‑Str. 2, 17489 Greifswald, Germany
2. Greifswald University Medical Center, Department of General, Visceral, Thoracic and Vascular Surgery, 17475 Greifswald, Germany
3. University Hospital Essen, Department of Dermatology, Venereology, and Allergology, University of Duisburg-Essen, 45122 Essen, Germany
4. Rostock University Medical Center, Clinic for Dermatology and Venereology, Strempelstr. 13, 18057 Rostock, Germany
5. Institute for Hygiene and Environmental Medicine, Walther-Rathenau-Str. 48, 17489 Greifswald, Germany
6. Center for Precision Medicine, Department of Medicine, University of Texas Health San Antonio, San Antonio, TX, USA

* contributed equally as corresponding authors, address correspondence to:

rajesh.gandhirajan@inp-greifswald.de and sander.bekeschus@inp-greifswald.de

**Keywords**: cancer; glutathione; kINPen; melanoma; plasma medicine

*Supplementary figures

**
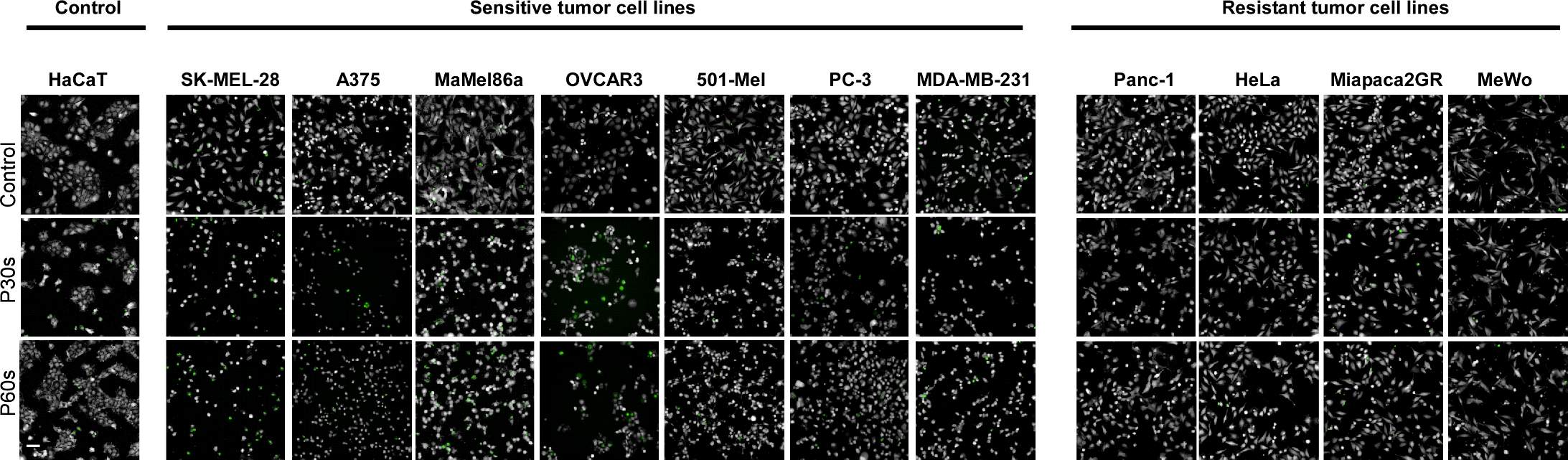
**

**Supplementary Figure 1.** ***Viability of cell lines determined using fluorescence microscopy.*** (A) Representative images form sytox green viability staining of ‘sensitive’ and ‘resistant’ tumor cell lines 24 hours after exposure to 30s (P30s) and 60s (P60s) of cold plasma treatment. Non-malignant HaCaT keratinocytes served as a non-tumorogenic control cell line. Scale bar: 50 µm.


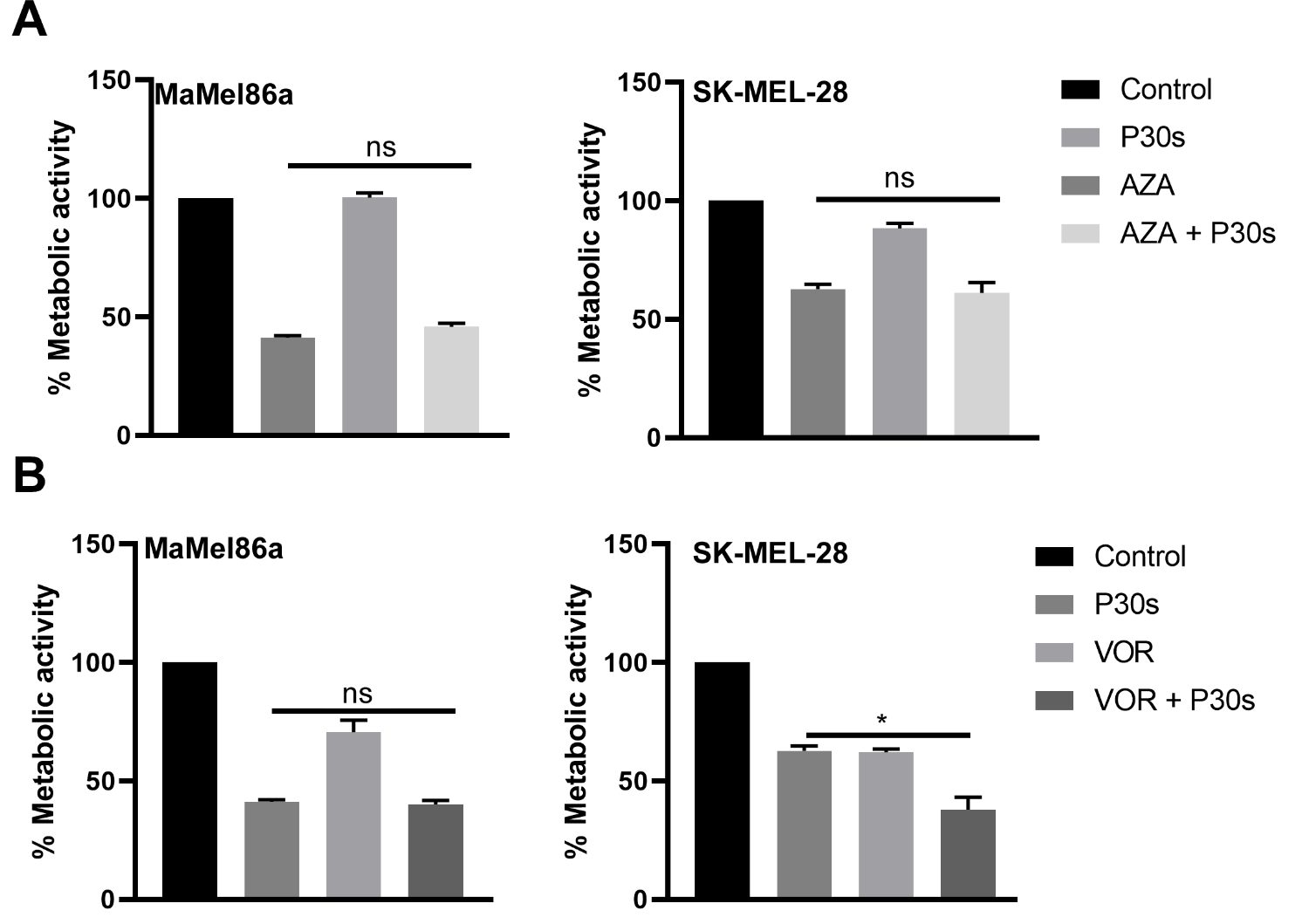


**Supplementary Figure 2. *Effect of* *DNA methylation, and histone deacetylation inhibitors on ‘sensitive’ cell lines in combination with cold physical plasma.*** (A) Metabolic activity of ‘sensitive’ cell lines upon pretreatment with DNA methylation inhibitor azacytidine (AZA; 5 μM). (B) Metabolic activity of ‘sensitive’ cell lines upon pretreatment with HDAC inhibitor vorinostat (VOR; 10 μM). All results are derived from three independent biological replicates, and results are shown as mean ±SEM. Statistical analysis was performed using Student's t-test with Welch's correction (B and C). ∗ = p < 0.05; ns = not significant.

**
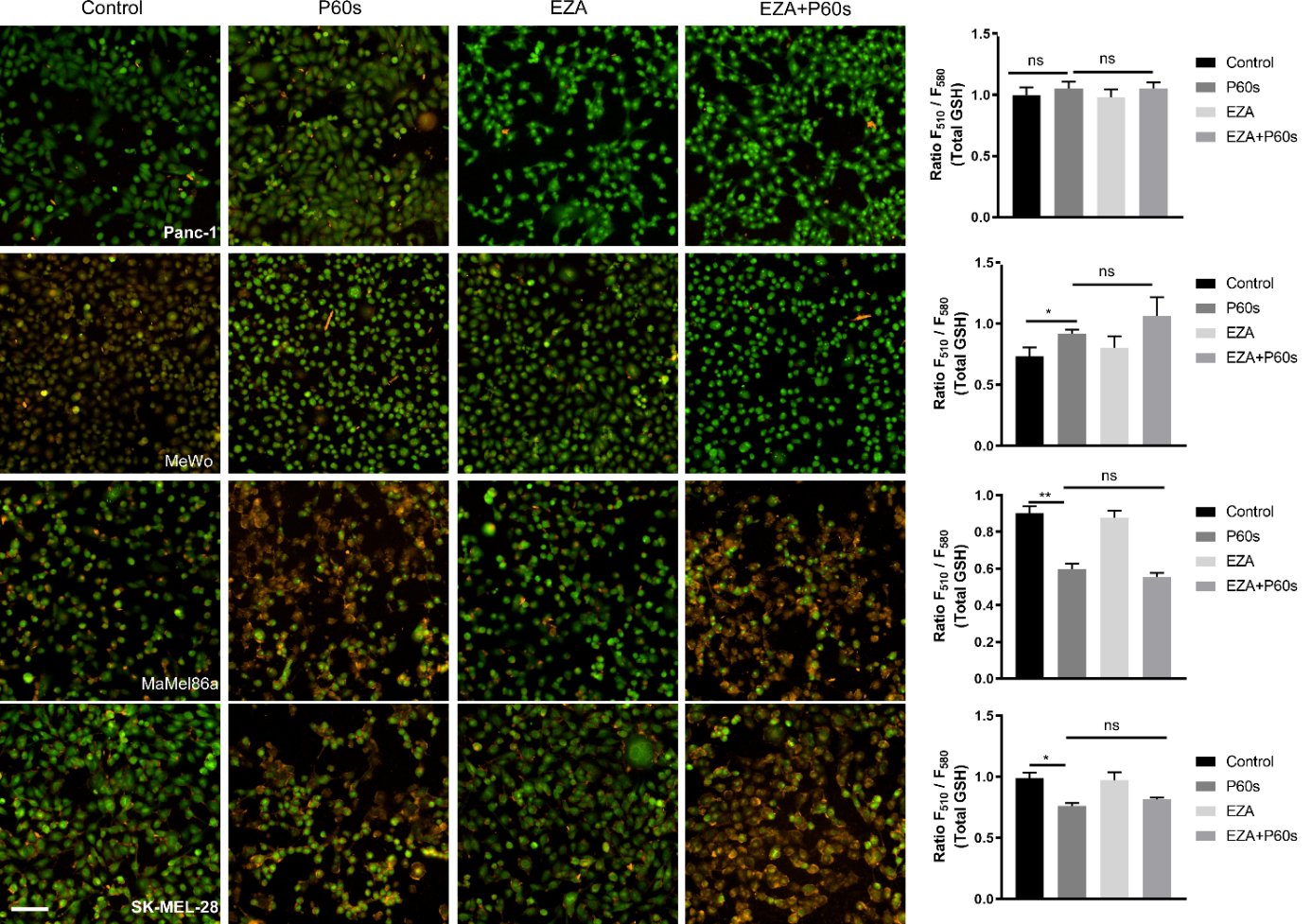
**

**Supplementary Figure 3. *Total GSH levels in ‘sensitive’ and ‘resistant’ cells following pre-treatment with GSTP1 inhibitor ezatiostat.*** (A) Representative images and quantification of GSHtracer in ‘resistant’ (MeWo and Panc-1) and ‘sensitive’(SK-MEL-28 and MaMel86a) pretreated with ezatiostat (EZA, 25 μM; 24 h) followed by exposure to 60 s of plasma treatment. Live cell imaging was performed six hours later with the green to orange ratio indicating total GSH levels in cells. Data are mean ±SEM from three independent experiments. Statistical significance was determined by Student's t-test with Welch's correction, * = p < 0.05; ** = p < 0.01; ns = not significant. Scale bar: 50 µm.

**
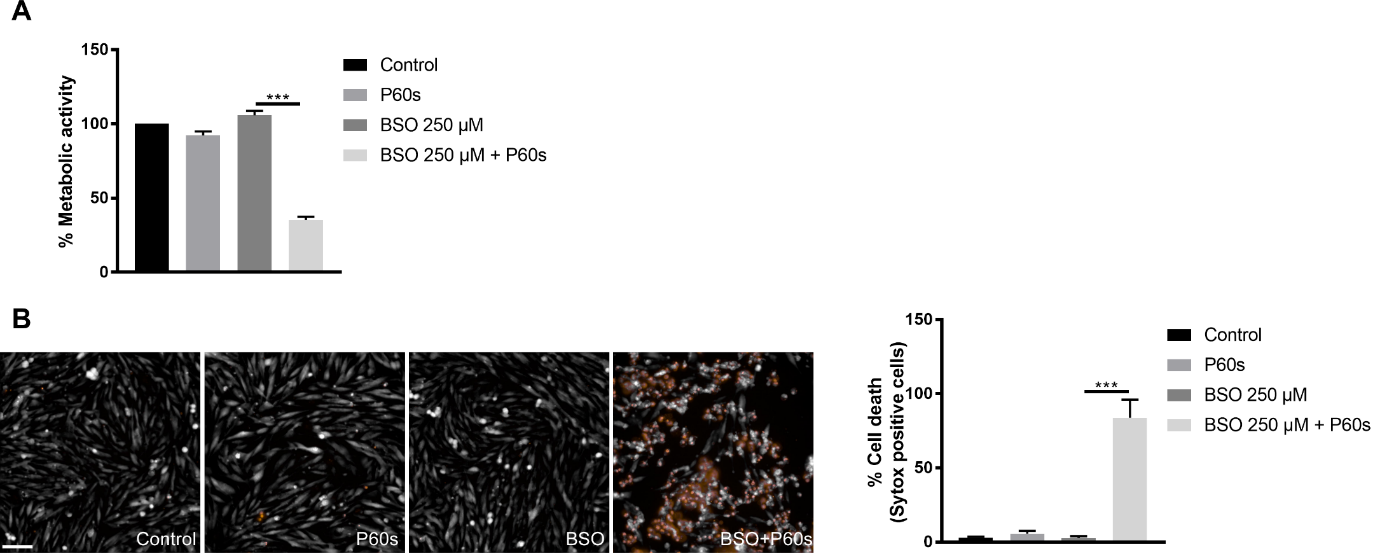
**

**Supplementary Figure 4. *Inhibition of γ-GCS sensitized resistant cells to cold plasma.*** (A) Metabolic activity of the ‘resistant’ cell line (MeWo) pretreated with Butathione sulfoximine (BSO, 500 μM; 24 h) followed by exposure to 60 s (P60s) of plasma treatment. (B) the ‘resistant’ cell line (MeWo) was treated with 250 µM of BSO for 24 h, followed by exposure to cold physical plasma and further incubation for 24 h before staining with sytox orange to determine the percent of terminally dead cells. Results are shown as mean +SEM. Statistical significance was determined by Student's t-test with Welch's correction, *** = p < 0.001. Scale bar: 50 µm.
